# The Dental Health of Orphan and Non-orphan Children in Fuyang City, China

**DOI:** 10.3290/j.ohpd.b2182947

**Published:** 2021-10-22

**Authors:** Jianbo Xu, Yuxun Zhu, Chundi Wang, Dandan Wang

**Affiliations:** a Dentist, Fuyang Hospital of Anhui Medical University, Fuyang, Anhui Province, China. Study design.; b Dentist, Fuyang Hospital of Anhui Medical University, Fuyang, Anhui Province, China. Performed the dental examinations.; c Dentist, Fuyang Hospital of Anhui Medical University, Fuyang, Anhui Province, China. Wrote the manuscript.; d Dentist, Fuyang Hospital of Anhui Medical University, Fuyang, Anhui Province, China. Performed the statistical analysis.

**Keywords:** dental health, DMF, epidemiology, orphan children

## Abstract

**Purpose:**

To assess the dental health of orphan and non-orphan children in Fuyang City, China.

**Materials and Methods:**

A total of 332 orphan children were selected from social child welfare institutes and 590 non-orphan children were selected from the Experimental Primary and Middle Schools through random sampling in Fuyang City, China. The indices for Decayed, Missing, Filling (DMF) in orphan and non-orphan children were determined by dental inspections to assess their dental health. The periodontal status of orphan and non-orphan children aged 12–15 years was determined using the percentages of bleeding gingiva and dental calculus.

**Results:**

In the age range of 3 to 5 years, the percentage of caries (81%) and the mean DMF (4.41; SD: 2.06; 95% CI: 3.82 to 5.00) of orphan children were statistically significantly higher than the percentage of caries (64%) and the mean DMF of the non-orphan control cohort (3.29; SD: 2.05; 95% CI: 2.87 to 3.71; p < 0.05). In the age range of 12 to 15 years, the percentage of caries (50.8%) and the mean DMF (1.28; SD: 1.26; 95% CI: 1.12 to 1.43) of orphan children were statistically significantly higher than the percentage of caries (34.5%) and the mean DMF (1.11; SD: 1.24; 95% CI: 1.01 to 1.23; p < 0.05) of non-orphan children. For orphan children ages 3 to 5 years, the percentage of restorations was statistically significantly lower (p < 0.05) than that of non-orphan children (30%). For orphan children ages 12 to 15 years, the percentage of restorations was 3.9%, statistically significantly lower (p < 0.05) than that of non-orphan children (19.4%). In orphan children ages 12 to 15 years, the percentages of bleeding gingiva (76.0%) and dental calculus (69.3%) were statistically significantly higher (p < 0.05) than those of the non-orphan children (46.2% and 39.1%, respectively). There was no statistically significant difference in the evaluation indicators above between different genders within the groups studied, except the percentage of restorations.

**Conclusion:**

The dental health of orphan children in Fuyang City is worse than that of non-orphan children of the same age ranges. The values determined in this study can be used as a starting metric to measure the effectiveness of dental health care programs in improving the dental health of both orphan and non-orphan children.

Orphans are in a disadvantaged social situation due to lack of parental support, and they are a vulnerable group that attracts social attention. Orphans who are guarded and educated by orphanages are separated from ordinary children in their daily life, lack the support of the family system of parents and relatives, and are isolated from mainstream society for a long time, so that they have obvious identity characteristics. Orphan children are typically raised in socioeconomic poverty, lack adequate healthcare, live outside affluent society, and experience psychological trauma.^4,7^ Maintaining dental health is difficult for orphan children because of their living environment, the lack of professional dental health care, the low level of dental health knowledge, and poor dental health habits.^2,6^ Studies have shown that school-aged orphan children in India are at a higher risk of caries and periodontal disease compared with similarly-aged non-orphan children.^12,16^ Dental health care is one of the most common unmet health needs of the orphaned population.^5,15,20^

The dental health of orphan and non-orphan children aged 3–5 and 12–15 years was investigated to provide baseline dental health data on orphan and non-orphan children in Fuyang City, China. The purpose was to gain understanding of the dental health management risks faced by orphan children, and further guide the follow-up dental health care measures for them.

## Materials and Methods

### Sample Size and Subjects

The study followed the survey requirements of the fourth China National Dental Health Survey^21^ and the fifth edition of WHO Oral Health Surveys Basic Methods.^19^ From September 15, 2020 to December 15, 2020, orphan children aged 3–5 and 12–15 years from social child welfare institutes and non-orphan children in the same age ranges from Experimental Primary and Middle Schools were selected through random sampling in Fuyang City.

Fuyang has a registered population of more than 10 million, of which a quarter are young and middle-aged migrant workers. The children ages 0–15 make up a quarter of the total population, and their supply of social public resources such as health care, pre-school education, and compulsory education is facing great pressure. Therefore, understanding the oral health of children in Fuyang is ultimately beneficial for their oral health care. We selected schools and orphanages in Fuyang City by lottery, and all children from the selected institutions were regarded as a whole. After all the children were numbered, random numbers were generated by a random number generators, and then the children corresponding to the random numbers were studied.

The sample size calculation formula:

$$n=\frac{U{\frac{\alpha }{2}}^{2}(1-p)}{\delta^{2}}$$

was used to estimate the sample content, where the estimated value p was 34.5% and 70.9%, respectively, for the prevalence of dental caries among children aged 5 and 12 years in the fourth China National Dental Health Survey. The inspection level α was set to two-sided 0.05, and the sampling error δ was set to 10%p. The theoretical sample sizes for the 3–5 and 12–15 age groups were 157 and 729, respectively. Finally, a stratified multi-stage random sampling method was used to actually sample 172 and 750 children of the corresponding age. Orphan children (n = 332) and non-orphan children of same age ranges (n = 590) were randomly selected. Gender was the only basic information recorded.

The DMF indices of primary teeth in children aged 3–5 years and permanent teeth in children aged 12–15 years were determined in both orphan and non-orphan groups. The inclusion criteria for orphan children were that the children had lived in social child welfare institutes for at least 3 years prior to the study and had received caretaker permission to participate in the survey. The inclusion criteria for non-orphan children were that the children were attending school and had obtained the approval of school leaders and teachers. All children with severe systemic diseases or disabilities, or taking long-term medications, were excluded from the study, regardless of their status (orphan/non-orphan).

This study was conducted with the consent and guidance of the Ethics Committee of Fuyang Hospital of Anhui Medical University. Our research was considered to be beneficial to the improvement of the oral, physical, and mental health of the orphan children in Fuyang by the ethics committee. The specific process of the research did not cause any physical or psychological harm to the children included.

### Clinical Examination

The percentages of caries and mean DMF indices were determined in all children. The percentage of teeth per child exhibiting of gingival bleeding and dental calculus was used to evaluate periodontal status in all children 12–15 years of age. Visual and exploratory examinations were performed using disposable oral examination trays containing forceps, plane oroscopes and CPI probes (Shanghai Kangqiao Dental Instruments Factory; Shanghai, China), as well as cotton balls. The screening and examination criteria were based on the fourth China National Dental Health Survey.^21^ Each child was examined by each of three separate specialists with epidemiological training. The standard consistency test results before the survey were reliable (Kappa value > 0.8). The procedure and instrumentation of the investigation were in accord with the fifth edition of WHO Oral Health Surveys Basic Methods.^19^

### Statistical Analysis

SPSS 22.0 (IBM; Armonk, NY, USA) was used for statistical analysis of the data. Measurement data are expressed as mean, standard deviation and 95% CI. An independent t-test was used to compare the means of the two samples. Enumeration data are expressed as percentages, and mean comparisons were performed using the chi-squared test and Fisher’s exact test. The significance level (α) was set to 0.05. p > 0.05 indicated no statistically significance difference.

## Results

Among the 332 orphan children, 23.5% were in the 3- to 5-year age group and 76.5% were in the 12- to 15-year age group. Among the 590 non-orphan children, 19.2% were in 3 to 5-year age group and 84.1% were in the 12- to 15-year age group.

The total percentage of caries in non-orphan children aged 3–5 years (64%) was statistically significantly lower (p < 0.05) than that of the orphan cohort (81%) (Table 1). There was no statistically significant difference in the percentages of caries between genders within the 3–5 age range of orphan and non-orphan children (Table 1). The total percentage of caries in non-orphan children aged 12–15 (34.5%) was statistically significantly lower (p < 0.05) than that of the orphan cohort (50.8%) (Table 1). There was no statistically significant difference in the percentages of caries between genders within the 12- to 15-year age range of orphan and non-orphan children (Table 1).

**Table 1:** Percentage of caries in orphan and non-orphan children

	Children aged 3–5 years (172)			
	Orphan (78)		Non-orphan (94)	
Caries (%)	81		64 *	
Condition	Male (43)	Female (35)	Male (53)	
Caries (%)	79	83 **	68	
	Children aged 12–15 years (760)			
	Orphan (254)		Non-orphan (496)	
Caries (%)	50.8		34.5 *	
Condition	Male (145)	Female (109)	Male (282)	Female (214)
Caries (%)	48.3	54.1 **	34.0	35.1 **

* p < 0.05. The percentage of caries in non-orphan children was statistically significantly lower than orphan children; ** p > 0.05, there was no statistically significant difference in percentage of caries between by gender.

In the 3- to 5-year age group, the mean DMF index of orphan children (4.41; SD: 2.60; 95% CI: 3.82 to 5.00) was statistically significantly higher (p < 0.05) than that of non-orphan children (3.29; SD: 2.05; 95% CI: 2.87 to 3.71) (Table 2). There were no statistically significant differences in the DMF indices between genders within the 3- to 5-year age range of orphan and non-orphan children (Table 2). In the 3- to 5-year age range, the mean DMF index of orphan children increased 34.0% over that of the non-orphan cohort. The upper limit of the 95% CI for non-orphan children (3.71) did not overlap with that of the lower limit of the 95% CI for orphan children (3.82).

**Table 2:** DMF constituents of orphan and non-orphan children

Condition	Children aged 3–5 years (172)			
	Orphan (78)		Non-orphan (94)	
	Male (43)	Female (35)	Male (53)	Female (41)
Dt	4.09	4.26	2.79	2.41
Mt	0.49	0.20	0	0
Ft	0.05	0.09	0.66	0.66
DMF, mean (SD)	4.37(2.70)	4.46 (2.51) **	3.45 (1.91)	3.07 (2.22) **
Total DMF, mean (SD) [95% CI]	4.41 (2.60) [3.82 to 5.00]		3.29 (2.05) [2.87 to 3.71] *	
Condition	Children aged 12–15 years (750)			
	Orphan (254)		Non-orphan (496)	
	Male (145)	Female (109)	Male ( 282)	Female (214)
Dt	154	127	218	158
Mt	13	10	2	1
Ft	11	9	83	91
DMF, mean (SD)	1.23 (1.28)	1.34 (1.23) **	1.07 (1.28)	1.17 (1.1) **
Total DMF, mean (SD)	1.28 (1.26) [1.12 to 1.43]		1.11 (1.24) [1.01 to 1.23] *	

* p < 0.05, the mean DMF index of orphan children was statistically significantly higher than that of non-orphan children; ** p > 0.05, there was no statistically significant difference of mean DMF index between genders.

In the age group 12–15 years, the mean DMF index of orphan children (1.28; SD: 1.26; 95% C: 1.12 to 1.43) was statistically significantly higher (p < 0.05) than that of non-orphan children (1.11; SD: 1.24; 95% CI: 1.01 to 1.23) (Table 2). There were no statistically significant differences in the DMF indices between genders within the age group 12–15 years of orphan and non-orphan children (Table 2). There was a 13.3% increase in the mean DMF index of orphan children compared to that of the non-orphan cohort. However, the 95% CI for the cohorts overlapped considerably.

The percent teeth with bleeding gingiva (76.0%) and dental calculus (69.3%) in orphan children was statistically significantly higher (p < 0.05) than that of non-orphan children (46.2% and 39.1%, respectively). There were no statistically significant differences in the percentages of bleeding gingiva and dental calculus between genders in orphan and non-orphan children (p > 0.05).

**Fig 1:**
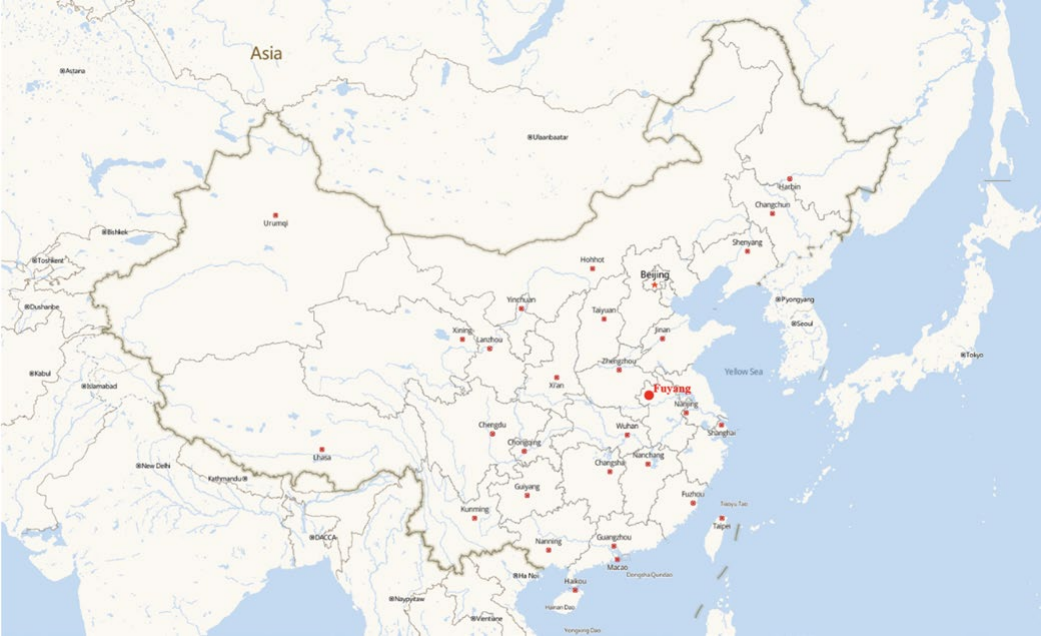
Location of Fuyang, China.

## Discussion

Most epidemiological studies on dental health have focused on non-orphan school-age children rather than on the dental health of orphan children,^10,11,13,20^ and as a result, orphan children have not often been the focus of dental health promotion.^1,8,22^ This study compared the caries percentages, DMF indices, restoration percentages, and periodontal health of orphan and non-orphan children aged 3–5 and 12–15 years in Fuyang City, China. A survey on the dental health of orphan children aged 4 to 17 in Chongqing, China, found that the percentages of caries in primary teeth and permanent teeth of orphan children were 50.0% and 39.5%, respectively, suggesting poorer dental health in orphan children vs non-orphan children, and that they need more comprehensive dental health care compared with that of non-orphan children in the same age in other studies.^15^ A study on the dental health of 360 primary- and middle-school orphan children in Jilin province found that the percentage of caries of orphan children was 67.2%.^5^ The percentage of caries of orphan children in our study was significantly higher than the results of the above two studies, which might be due to the low level of incomes and expenditures of the urban population in Fuyang. However, unlike our study, the above two studies did not involve a survey of non-orphan children. Moreover, these and other studies concluded that the lack of dental health knowledge and poor hygiene habits had led to the poor oral health of orphan children.^3,5,9,14,15,17,18^

In this study, the percentage of caries, mean DMF, percentage of restorations demonstrated significantly worse dental health of orphans compared to non-orphans in both the 3- to 5-year and 12- to 15-year age ranges. These results suggested that orphans in Fuyang suffer from poorer medical and health conditions, less dental health care, less dental health education, and ineffective and low-quality diagnosis and treatment of oral diseases.

The caries percentages of orphan and non-orphan children found in this study is higher than those of the same age children in the fourth China National Dental Health Survey (3–5 years old, 62.5%;12–15 years old, 41.9%).^21^ Moreover, as a labor-exporting city, many school-age children in Fuyang city are ‘left behind’ children whose parents work abroad all year round. Due to the lack of parental guidance on oral health, dental health problems in these children could not be adequately resolved, delaying the diagnosis and treatment of oral diseases. The periodontal status of children aged 12–15 years showed that the percentages of gingival bleeding and dental calculus were higher in orphan children than those of non-orphan children. The maintenance of periodontal health was an issue for both groups of children, which could be addressed by an improvement in the periodontal health education of all workers in the field of stomatology.

The results of our research provide basic data that can be used for comparison in the follow-up dental health survey of orphan children. Clear knowledge has been gained on which specific indicators of the dental health of orphan children can be improved.

It should be emphasised that the population investigated in this study was only orphan children from social child welfare institutes and students from two schools, which may not fully reflect the dental health of orphan children and non-orphan children in Fuyang city. Therefore, a more extensive and representative oral epidemiological investigation in Fuyang is needed to increase the attention paid to the dental health of orphan children.

## Conclusion

The dental health of orphan children in Fuyang is significantly worse than their non-orphan counterparts, which indicates that the dental health promotion of orphan children is insufficient. The results of this study can be used as initial metrics to evaluate the effectiveness of various dental health care programs in improving the dental health of both orphan and non-orphan children. Our department will set up an oral health promotion team for orphan children to conduct regular oral health surveys. Through the comparison of the results of surveys, we will intervene for specific factors. At the same time, we will carry out oral health education for different age groups to help them develop good oral habits.
